# Investigation and Rapid Discrimination of Food-Related Bacteria under Stress Treatments Using IR Microspectroscopy

**DOI:** 10.3390/foods10081850

**Published:** 2021-08-11

**Authors:** Daniel Klein, René Breuch, Jessica Reinmüller, Carsten Engelhard, Peter Kaul

**Affiliations:** 1 Institute of Safety and Security Research, Bonn-Rhein-Sieg University of Applied Sciences, Von-Liebig-Straße 20, 53359 Rheinbach, Germany; rene.breuch@h-brs.de (R.B.); reinmuellerje@aol.com (J.R.); peter.kaul@h-brs.de (P.K.); 2 Department of Chemistry and Biology, University of Siegen, Adolf-Reichwein-Str. 2, 57076 Siegen, Germany; engelhard@chemie.uni-siegen.de; 3 Research Center of Micro- and Nanochemistry and (Bio)Technology, University of Siegen, Adolf-Reichwein-Str. 2, 57076 Siegen, Germany

**Keywords:** IR microspectroscopy, food-related bacteria, classification, discriminant analysis, stress response, food safety, chemometrics

## Abstract

Because the robust and rapid determination of spoilage microorganisms is becoming increasingly important in industry, the use of IR microspectroscopy, and the establishment of robust and versatile chemometric models for data processing and classification, is gaining importance. To further improve the chemometric models, bacterial stress responses were induced, to study the effect on the IR spectra and to improve the chemometric model. Thus, in this work, nine important food-relevant microorganisms were subjected to eight stress conditions, besides the regular culturing as a reference. Spectral changes compared to normal growth conditions without stressors were found in the spectral regions of 900–1500 cm^−1^ and 1500–1700 cm^−1^. These differences might stem from changes in the protein secondary structure, exopolymer production, and concentration of nucleic acids, lipids, and polysaccharides. As a result, a model for the discrimination of the studied microorganisms at the genus, species and strain level was established, with an accuracy of 96.6%. This was achieved despite the inclusion of various stress conditions and times after incubation of the bacteria. In addition, a model was developed for each individual microorganism, to separate each stress condition or regular treatment with 100% accuracy.

## 1. Introduction

Because meat and meat products are highly appreciated by consumers, for their nutritional value and taste, the global supply of meat is expected to continue to increase in the coming years [1]. However, meat is highly prone to microbial spoilage and, therefore, rapid and easy identification of contamination is a major concern in food safety [1,2]. This will help to ensure measures to minimize health hazards, and thus prevent foodborne illness and unnecessary food waste along the supply chain [2].

However, as bacteria are subject to constant fluctuations in their growth conditions, both in nature and along the supply chain, they have developed capabilities to constantly adapt to conditions, or even change to a state of viability, but are non-cultivable [3,4,5]. This makes sub-lethally damaged cells difficult to detect with classical laboratory culture techniques [2]. Additionally, standard methods, such as classical microbiology, sensory-mechanical studies, and immunological or genetic techniques, have disadvantages in speed, complexity, and invasiveness [1,6,7,8,9]. However, these viable, but non-culturable, microorganisms can be revived within the supply chain, and thus not only affect the product’s usability, but may also be a health hazard [2,9,10,11].

Infrared (IR) spectroscopy has been successfully used to detect and identify microorganisms [10,12,13,14]. In recent years, many studies dealt with the IR spectroscopic evaluation of specific effects of stress conditions on microorganisms, such as protein misfolding [15], phase behavior of the cell membranes of *Escherichia coli* (*E. coli*) during desiccation, rehydration, and growth recovery [16,17], or the sonication injury on *Listeria monocytogenes* [18]. Moreover, IR spectroscopy was used to study the influence of nanoparticles on *E. coli* [19,20], and the effects of heavy metals on *Brevundimonas* sp., *Gordonia* sp., and *Microbacterium oxydans*, using the analysis of variance, hierarchical cluster analysis, principal component analysis (PCA), and soft independent modelling of class analogies (SIMCA) [21,22]. Additionally, the influence of heat on *Lactococcus lactis*, *Salmonella enterica*, and *Listeria monocytogenes* was evaluated by the analysis of the IR peak area of amide I and amide II bands, and the extent of injury was predicted by the analysis of the wavenumber area of 900–1300 cm^−1^ by SIMCA and partial least squares regression analysis (PLSR) [23,24]. Furthermore, the response of *E. coli*, *Campylobacter jejuni*, and *Pseudomonas aeruginosa* that were exposed to cold- [25,26], chemical- [25] and pH-stressors [25,27,28] was studied by DNA microarrays and Fourier-transform (FT) IR analysis, coupled to PCA, discriminant function analysis, and PLSR. 

While interesting findings have been reported, IR microspectroscopy, in combination with PCA and canonical discriminant analysis, has not been used so far, to combine different stress conditions on numerous food-related microorganisms at different times after incubation, in one chemometric model.

The food industry is particularly interested in the following most dominant microorganisms that are detected on fresh and chilled meat, and other food products: *Pseudomonas spp.*, especially *Pseudomonas fluorescens* (*Ps. fluor*) and *Enterobacteriaceae*, such as *E. coli*, *Micrococcus luteus* (*M. luteus*), *Bacillus thuringiensis israelensis* (*B. tii*), *Bacillus coagulans* (*B. coag*), *Bacillus subtilis* (*B. sub*), and *Brochothrix thermosphacta* (*B. therm*) [29,30,31,32,33,34,35,36].

Therefore, the aim of this study was the development of a rapid and non-destructive analysis method for food-related microorganisms. The influences of numerous stress-conditioned microorganisms, as well as regularly treated microorganisms, over various aging stages on agar plates, were analyzed with IR spectroscopy, to build up an extensive data set. Chemometric models were developed to discriminate the influence of stress, and also to discriminate the selected microorganisms independent of their stress conditions within one model down to the strain level.

## 2. Materials and Methods

### 2.1. Bacterial Cultures and Sample Preparation

The following nine microorganisms were cultivated on nutrient agar (10 g/L meat peptone, 10 g/L meat extract, 5 g/L sodium chloride and 18 g/L agar-agar (Merck KGaA, Germany)) and prepared according to our previously published method [12,37,38]: *Bacillus subtilis* DSM 10, *Bacillus coagulans* DSM 1, *Escherichia coli* K12 DSM 498, *Escherichia coli* TOP10, *Micrococcus luteus* DSM 20030, *Brochothrix thermosphacta* DSM 20171, *Pseudomonas fluorescens* DSM 4358, *Pseudomonas fluorescens* DSM 50090 and *Bacillus thuringiensis israelensis* DSM 5724.

As described in our previously published methods [12], the samples were taken by a blotting technique with the sample carrier (stainless steel cylinder) directly from the agar plate without any sampling pre-treatments (e.g., centrifugation, washing, drying). IR spectra were recorded directly (lifetime conditions) after sampling or directly after the stress impact (sampling condition) without any incubation period after sampling. Detailed information can be found in Section 2.2.

The spectral data set of each microorganism, consisting of four independent data sets for each stress condition, were divided into independent training and test data sets. Further information can be found in Section 2.4. 

### 2.2. Sample Treatment

In order to expose the microorganisms to different influences, they were subjected to lifetime conditions (incubation under acidic and alkaline conditions, incubation at different temperatures and incubation under 2-propanol influence) and sampling conditions (cold sampling, heat sampling and desiccation) in a controlled manner, in addition to the regular reference treatment. Incubation was performed for all microorganisms in a Binder BD 240 (BINDER GmbH, Tuttlingen, Germany) incubator.

#### 2.2.1. Reference Samples (Regular Treatment)

All microorganisms were cultivated in accordance to DSMZ (Leibniz Institut DSMZ—German Collection of Microorganisms and Cell Cultures, Braunschweig, Germany) guidelines. These samples served as reference samples in this study.

#### 2.2.2. Incubation under Acidic Conditions

To expose microorganisms to acidic pH stress, a hydrochloric acid (HCl) solution (36%, Alfa Aesar, Ward Hill, MA, USA) with pH 1 (verified by means of pH indicator paper, Th. Geyer GmbH & Co. KG, Renningen, Germany) was prepared. The agar plates were completely covered with the hydrochloric acid solution (2 mL) and the inoculation took place onto the hydrochloric acid-covered agar plates. Afterwards the cultivation was performed in accordance to DSMZ guidelines.

#### 2.2.3. Incubation under Alkaline Conditions

Complementary to the incubation under acidic conditions, a sodium hydroxide solution (sodium hydroxide pellets, Merck, Darmstadt, Germany) (pH 13 (verified by means of pH indicator paper)) was prepared to expose microorganisms to alkaline pH stress. Afterwards, the cultivation was performed in accordance to DSMZ guidelines.

#### 2.2.4. Incubation at Lower/Higher Temperatures

Microorganisms were incubated at a temperature of 25 °C and 45 °C.

#### 2.2.5. Incubation with 2-Propanol

Complementary to the incubation under acidic and alkaline stress, the microorganisms were stressed with 2-propanol (99.9%, Höfer Chemie GmbH, Kleinblittersdorf, Germany). Afterwards the cultivation was performed in accordance to DSMZ guidelines.

#### 2.2.6. Cold Sampling

Microorganisms were sampled from regular treated samples, covered with liquid nitrogen for 60 s and instantly measured.

#### 2.2.7. Heat-Drying

Microorganisms were sampled from regular treated samples, dried at 50 °C for 60 min and instantly measured.

#### 2.2.8. Desiccation

Microorganisms were sampled from regular treated samples, dried in a desiccator filled with silica gel for 60 min and instantly measured.

### 2.3. Instrumentation

The samples were examined in reflectance mode, 20 scans per spectrum, 4 cm^−1^ resolution and 20× magnification (Cassegrain objective (Bruker Ser.910/1022346, numerical aperture: 0.6, working distance: 6 mm) by means of a Hyperion 3000/Vertex 70 Fourier-transform IR microspectrometer (Bruker Optics GmbH, Ettlingen, Germany) with mercury/cadmium/telluride (MCT) detector. Instrument controlling and data acquisition was carried out by the OPUS 7.5 software (Version 7.5, Ettlingen, Germany). 

Due to the microscopic component, the morphological properties of heterogeneous samples can be combined with spectral data and samples, with a few hundred microorganisms being determined [39,40]. This often results in no further sample preparation than a transfer of the sample to a sample carrier [41]. In addition, the required analysis time is reduced compared to classical IR spectroscopy [40].

### 2.4. Data Handling and Visualization

IR spectra were subsequently sum normalized (OriginPro 2019b, OriginLab Corporation, Northampton, MA, USA), data reduced to the range of 915–1750 cm^−1^ and 2825–3680 cm^−1^ to exclude the characteristic CO_2_ region and the lower fingerprint area, the first derivative was built and smoothed with a 13-point Savitzky–Golay filter (LabVIEW 2016; National Instruments, Austin, TX, USA). 

**Figure 1:**
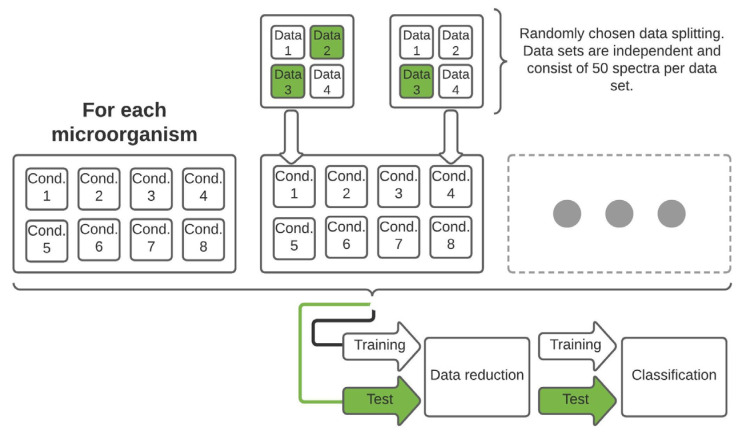
Schematic representation of the data set structure as well as the splitting process with subsequent data reduction and classification. The training and test data set contains the corresponding information of each measured microorganism and thus of each growth and sampling condition.

The splitting for training and test data was carried out so that one or two independent data sets, each with 50 spectra for each stress condition and regular treatment, was used as test data (Figure 1). 

The splitting process of training and test data is depicted in Table 1. Detailed information about the exact splitting pattern, the time after incubation of the microorganisms at the time of measurement and thus how long the microorganisms were exposed to the lifetime stress conditions can be found in the Table S1 (Supplementary Material). 

Because balanced training data sets are important, not only for data reduction by PCA, but also for robust, reliable and unweighted model development [42,43], the data sets were split into training and test data in different ways for the reason that not all stress conditions could be measured for all bacteria (see Section 3). 

For the following data evaluation, principal component analysis (PCA) was applied to the training data; the test data were converted into the vector space of the training data and the data were classified by a canonical discriminant analysis (CDA) by means of LabVIEW 2016 and OriginPro 2019b.

## 3. Results and Discussion

**Figure 2:**
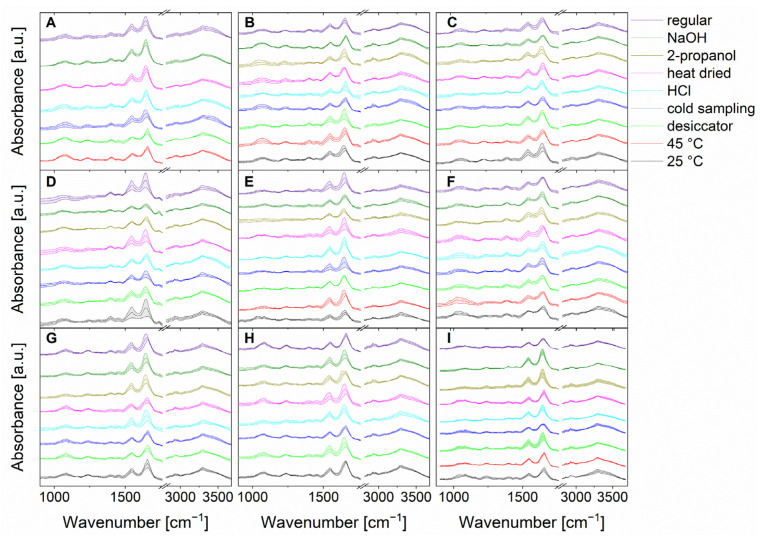
Stacked mean IR spectra of the normalized data of in total 15,200 spectra (200 spectra of each stress condition and regular treatment) of *B. coag* (**A**), *B. sub* (**B**), *B. therm* (**C**), *B. tii* (**D**), *E. coli* K12 (**E**), *M. luteus* (**F**), *Ps. fluor* 4358 (**G**), *Ps. fluor* 50090 (**H**) and *E. coli* TOP10 (**I**). Standard deviations are indicated by color-coded bands around the mean value.

First, the microorganisms were exposed to the different bacterial stress conditions mentioned above, and IR microspectroscopic data were carefully acquired. Figure 2 shows the mean IR spectra, including their standard deviations of bacteria under normal culture conditions versus bacteria under stress conditions. It is important to note that for *B. coag* no bacterial growth was detected at 25 °C, and when 2-propanol was used. Also, *B. tii* and *Ps. fluor* did not grow at 45 °C. This suggests that these stress conditions lead a non-culturable state. As a result, these conditions are not shown in Figure 2, and cannot be used for further evaluation.

Because spectral differences between individual microorganisms and individual stress conditions are based on different compositions in proteins, nucleic acids, lipopolysaccharides, or lipids of the cell, visual discrimination of 15,200 spectra in total (Figure 2) is almost impossible [10]. The fine spectral differences are in the range of the P=O vibrations of phospholipids (1085 cm^−1^ and 1240 cm^−1^) and the C–O–C vibrations in polysaccharides (900–1200 cm^−1^) [10,40]. In addition, differences can be noted in the C=O, C–H, and C–O–H vibrations of fatty acids and proteins [10,40]. In the area of proteins, strong bands of amide I and amide II vibrations (1550–1675 cm^−1^) can also be observed [10,40]. Furthermore, various C–H and N–H stretching vibrations from fatty acids and proteins can be identified in the range of 2850 cm^−1^ [10,40]. Here, chemometric approaches can aid in the classification and were carefully optimized, as discussed below. 

### 3.1. Bacteria Prediction Model

To ensure optimal model development and to avoid overfitting (performance plot: Figure S1 (Supplementary Material), the first 20 PCs (Figures S2 and S3) were used for model building using discriminant analysis.

As the covariance matrices of the training data classes had no significant equality, a quadratic, instead of a linear discriminant function, was chosen [44,45,46].

The error for classification and cross-validation of the training data was 0.01%; one spectrum of *E. coli* K12 was assigned to *E. coli* TOP10. In order to test the developed model for the classification of food-related microorganisms for robustness, accuracy, and reproducibility, independent test data sets of the trained classes were added to the model.

**Figure 3:**
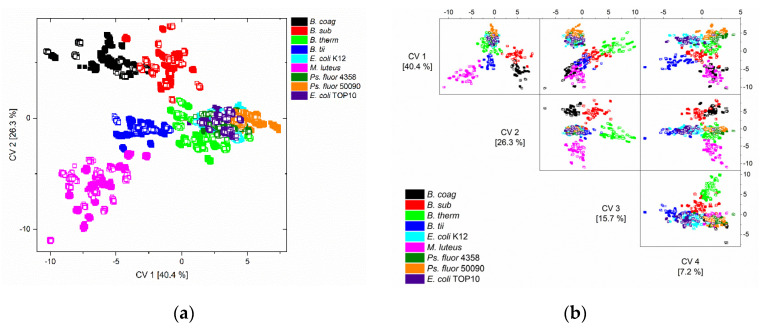
Scatter (**a**) and scatter matrix (**b**) plot of canonical variable one and two, and one to four of the QDA of the training data (solid squares) and the independent test data (unfilled squares) of all nine microorganisms and all nine conditions.

The first two (a) and the first four (b) canonical variables (CV) of the quadratic discriminant analysis (QDA) of the training data (solid squares) and test data (unfilled squares) are depicted in Figure 3a. On closer examination, it is noticeable that the test data are located exactly in the space of the training data.

*M. luteus* and all *Bacillus* spp. (*B. coag*, *B. sub*, *B. therm*, *B. tii*) could be separated from each other by the first two canonical variables, and *Pseudomonas fluorescens* from *E. coli* could be separated by the first four canonical variables (Figure 3b).

In conclusion, the data of the classification of the independent test data are presented in a confusion matrix (Table 2), which gives the number of spectra that were classified to the correct (diagonal) or wrong predicted class.

It can be observed that the classification of the test data, and thus the model development of a robust and meaningful model, was successful. The error rate of the classification of the independent test data was only 3.4%, and can be found in the classes of *B. coag*, *E. coli* K12 and TOP10 and *Ps. fluorescens*. 

Because the complete dataset consists of a large number of sub-datasets per microorganism, a detailed analysis of the classification errors on the sub-datasets is given in Table 3.

The detailed analysis of the error rate shows that a major part of the error was due to the misclassification of *E. coli* K12 to TOP10, and vice versa, and the assignment of *Ps. fluorescens* 4358 to *Ps. fluorescens* 50090. In addition, another part of the misclassification was due to the assignment of *E. coli* K12 to *Ps. fluorescens*. 

These misclassifications were also indicated by the graphical representations (Figure 3) of the classification results, where it is at least visually apparent that the distinction between the respective *E. coli* and *Ps. fluorescens* strains, as well as the separation between *E. coli* and *Ps. Fluorescens*, appears difficult. However, the detailed error analysis shows that the separation between the *Ps. fluorescens* strains was feasible, but the separation between the *E. coli* strains was more difficult. 

In contrast to other approaches, the presented results for the general discrimination of food-related bacteria were carried out with 20 scans per sample, as comparatively short measurement times [15,19,21,22,23,41,47] and in the absence of further sample preparation steps [15,18,21,22,25,26,47]. Nonetheless, the results demonstrate a non-inferior classification, even compared to macroscopic and microscopic studies, in which standardized work was performed in other approaches [12,23,26,41,48,49,50]. The novel aspect of this model, in comparison to the literature, is the inclusion of numerous stress conditions on microorganisms and the consideration of these stress conditions on numerous bacteria in one model, because often only the influence of a few stress conditions on single or a few selected microorganisms were investigated [19,21,22,23,26].

### 3.2. Stress Condition Prediction Model

Because clusters within a class (microorganisms) were visible, but do not seem to significantly influence the model for the discrimination of food-related microorganisms, it was reasonable to investigate what general influence the stress conditions have on the bacteria, or on the model. Therefore, we tested whether this type of model was also able to separate different stress conditions from each other.

All data were preprocessed, as already described for the discrimination model. The model building for the discrimination of different stress conditions per microorganism was executed as in the previously described model. For this purpose, the fourth of the independent data sets for each stress condition was used as the test data, and the first three independent data sets were used as the training data.

**Figure 4:**
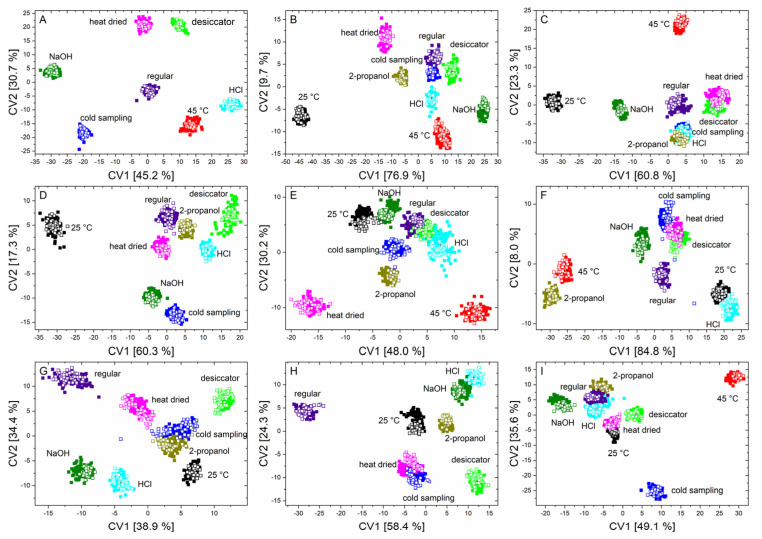
Scatter plots of canonical variable one and two of the QDA of the training data (solid squares) and the independent test data (unfilled squares) of *B. coag* (**A**), *B. sub* (**B**), *B. therm* (**C**), *B. tii* (**D**), *E. coli* K12 (**E**), *M. luteus* (**F**), *Ps. fluor* 4358 (**G**), *Ps. fluor* 50090 (**H**) and *E. coli* TOP10 (**I**) to discriminate all nine conditions.

The summary of the quadratic discriminant analyses of each microorganism is depicted in Figure 4.

The first two canonical variables clearly separate almost all the stress conditions for each microorganism. Furthermore, it was also evident that the test data can again be found exactly in the data clouds of the training data. 

The corresponding confusion matrices (Tables S2–S10 in the Supplementary Material) show that, for each microorganism, an error-free classification of the test data of each stress condition was possible, and all stress conditions were located in mostly isolated data clouds.

It can be observed that stressed microorganisms, in comparison to regularly treated microorganisms, show altered signals in the range of nucleic acids, polysaccharides, lipids, and in the region of –CH2/–CH3 stretching vibrations. Additionally, in most cases, a shift in the peak position was noticeable in the spectral region that was assigned to proteins (amide I and amide II vibrations) (Figure S4 (Supplementary Material)). Particularly notable were features such as those in Figure 4, where incubation at 25 °C for *B. sub* (B) causes this point cloud to be far removed from all other influencing conditions. This is because, in this case, the above-mentioned peaks have an increased intensity compared to regularly treated bacteria. It was also noticeable that, in the region of the amide I vibration, there is a shift to smaller wavenumbers. Observations of this nature were also found during stress reactions of the other microorganisms. For example, the data from *B. therm* (C), stressed with 2-propanol and HCl, cluster together as a result of a negative shift of the amide I band in both cases. Furthermore, a reduction in the bands in the fatty acid region was generally detected in *B. tii*, and desiccation of *E. coli* K12 only results in marginal changes in the spectrum (Figure S3 (Supplementary Material)). In addition, the heat drying of *E. coli* TOP10 results in a change in the ratio of nucleic acids, phospholipids, and polysaccharides. *M. luteus* shows significant changes in the ratio of lipids, nucleic acids, and proteins at incorrect incubation temperatures, and under NaOH and 2-propanol influence. Additionally, the two *Pseudomonas* species behave largely similarly under stress, but tend to undergo an opposite shift, *Ps. fluor* 4358 to higher and *Ps. fluor* 50090 to lower wavenumbers, in the amide I band (Figure S3 (Supplementary Material)).

For the range of 2800–3000 cm^−1^, our observations were consistent with the findings of Saulou et al. and Loffhagen et al., who presented that the spectra of viable microorganisms did not shift to lower wavenumbers, thus the microorganisms did not alter their membrane fluidity, but continued to show the presence of unsaturated bonds in lipids [19,51]. Furthermore, our results confirm the findings that stressed microorganisms show changes in the region of amide bands as part of their stress response mechanisms, indicating the alteration of the proteins secondary structure [19,23]. In addition, with the changes in the range of nucleic acids, polysaccharides, and lipids, resulting from the denaturation of nucleic acids, the production of exopolymer and effects on polysaccharides of the cell wall, in the range of 900–1300 cm^−1^_,_ could be confirmed [21,23,26,27].

In summary, the spectral changes of sub-lethally stressed microorganisms, such as the change in the ranges 900–1500 cm^−1^ and 1500–1700 cm^−1^, which indicate an altered concentration of nucleic acids, lipids, polysaccharides, as well as the shift of the amide bands, indicated by a change in the secondary structure of the proteins, are reproducible and extensively described in the literature mentioned before, for living or stressed microorganisms. Thus, it can be stated that a rapid, robust and meaningful model for the discrimination of food-related microorganisms down to the strain level, irrespective of sample age, lifetime stress conditions, and sampling stress conditions, could be established.

## 4. Conclusions

The response of food-related bacteria to stress gives rise to changes in their spectral features in FT-IR. Specifically, a method using simple sample preparation, fast measurement by IR microspectroscopy, and chemometrics, was carefully developed for the rapid and non-destructive analysis of food-relevant bacteria, independent of their time after incubation, cultivation conditions, and sampling condition. Classification, using canonical discriminant analysis, showed that a robust and meaningful model was developed to discriminate nine different microorganisms at the genus, species, and strain levels, with 96.6% accuracy. Furthermore, it was demonstrated that sub-lethally stressed microorganisms, irrespective of the lifetime or sampling condition, showed changes in the spectral range associated with nucleic acids, polysaccharides, lipids, –CH2/–CH3 stretching vibrations, and especially in the range of proteins (amide I and amide II vibrations), compared to reference microorganisms that were grown under well-established guidelines. These spectral changes were discussed and could indicate, for example, changes in the secondary structure of proteins and the production of the exopolymer.

The results obtained not only confirm the potential of IR microspectroscopy for the rapid differentiation of microorganisms and elucidation of the stress response of bacteria, but also show that the existing highly standardized databases should be expanded to include stress conditions, and reconsidered in terms of sample preparation and spectra quality. Continuing this approach, these models should be progressively supplemented by, for example, food samples, in order to take into account the influence of food matrices to the models.

## Figures and Tables

**Table 1:** Training and test data set sizes and data splitting ratio for the trained and tested microorganisms.

Class	Training Data	Test Data	Training Data [%]
*B. coag*	1050	350	75.0
*B. sub*	1050	750	58.3
*B. therm*	1050	750	58.3
*B. tii*	1050	550	65.6
*E. coli* K12	1050	750	58.3
*M. luteus*	1050	750	58.3
*Ps. fluor* 4358	1050	550	65.6
*Ps. fluor* 50090	1050	550	65.6
*E. coli* TOP10	1050	750	58.3

**Table 2:** Confusion matrix for the independent test data set. All nine conditions were pooled as one class per microorganism. The rows show the observed groups and the columns show the predicted groups. The values in the diagonal of the table reflect the correct classifications of observations into groups.

	Predicted Class								
Class	*B. coag*	*B. sub*	*B. therm*	*B. tii*	*E. coli* K12	*E. coli* TOP10	*Ps. fluor* 4358	*Ps. fluor* 50090	*M. luteus*
*B. coag*	**349**	1	0	0	0	0	0	0	0
*B. sub*	0	**750**	0	0	0	0	0	0	0
*B. therm*	0	0	**750**	0	0	0	0	0	0
*B. tii*	0	0	0	**550**	0	0	0	0	0
*E. coli* K12	0	0	0	0	**601**	99	0	50	0
*E. coli* TOP10	0	0	0	0	10	**740**	0	0	0
*Ps. fluor* 4358	0	0	0	0	1	11	**514**	24	0
*Ps. fluor* 50090	0	0	0	0	0	0	0	**550**	0
*M. luteus*	0	0	0	0	0	0	0	0	**750**

**Table 3:** Detailed analysis of the classification errors of the independent test data set on the sub-dataset level. The numbers are the total numbers of misclassified spectra in the specific sub-dataset.

Error Distribution	*B. sub*	*E. coli* K12	*E. coli* TOP10	*Ps. fluor* 50090
*B. coag*-HCl	1	--	--	--
*E. coli* K12-heat dried	--	--	49	--
*E. coli* K12-2-propanol	--	--	--	50
*E. coli* K12-regular	--	--	50	--
*E. coli* TOP10-heat dried	--	1	--	--
*E. coli* TOP10-regular	--	9	--	--
*Ps. fluor* 4358-cold sampling	--	--	8	--
*Ps. fluor* 4358-2-propanol	--	--	3	24
*Ps. fluor* 4358-NaOH	--	1	--	--

## Data Availability

The data presented in this study are available on request from the corresponding author.

## Supplementary Materials

The following are available online at https://www.mdpi.com/article/10.3390/foods10081850/s1, Table S1: Data splitting scheme for the trained and tested microorganisms. The time period after incubation is given in days for each data sets. Each data set consists of 50 spectra. Stress conditions are divided into lifetime conditions, in which the influence is already applied at inoculation and thus active over a life cycle, and sampling condition, in which the influence is a short major stress during sampling; Figure S1: Check for overfitting for the general discrimination of food-related microorganisms; Figure S2: Loadings (PC1–PC9) of the PCA of the training data set for the bacteria discrimination model. For a better spectral comparison in the graphs for PC1–PC3 the average spectrum of *B. coag* is given in gray; Figure S3: Loadings (PC10–PC20) of the PCA of the training data set for the bacteria discrimination model. For a better spectral comparison in the graphs for PC10–PC12 the average spectrum of *B. coag* is given in gray; Figure S4: Raw IR spectra of each stress condition vs. regular treated of *B. coag* (A), *B. sub* (B), *B. therm* (C), *B. tii* (D), *E. coli K12* (E), *M. luteus* (F), *Ps. fluor 4358* (G), *Ps. fluor 50090* (H) and *E. coli TOP10* (I); Table S2: Confusion matrix for the independent test data set for the classification of all stress condition for *B. coag*. The values of the table reflect the correct classifications of observations; Table S3: Confusion matrix for the independent test data set for the classification of all stress condition for *B. sub*. The values of the table reflect the correct classifications of observations; Table S4: Confusion matrix for the independent test data set for the classification of all stress condition for *B. therm*. The values of the table reflect the correct classifications of observations; Table S5: Confusion matrix for the independent test data set for the classification of all stress condition for *B. tii*. The values of the table reflect the correct classifications of observations; Table S6: Confusion matrix for the independent test data set for the classification of all stress condition for *E. coli* K12. The values of the table reflect the correct classifications of observations; Table S7: Confusion matrix for the independent test data set for the classification of all stress condition for *M. luteus*. The values of the table reflect the correct classifications of observations; Table S8: Confusion matrix for the independent test data set for the classification of all stress condition for *Ps. fluor* 4358. The values of the table reflect the correct classifications of observations; Table S9: Confusion matrix for the independent test data set for the classification of all stress condition for *Ps. fluor* 50090. The values of the table reflect the correct classifications of observations; Table S10: Confusion matrix for the independent test data set for the classification of all stress condition for *E. coli* TOP10. The values of the table reflect the correct classifications of observations.
