# Supplementary material for: Investigation and Rapid Discrimination of Food-Related Bacteria under Stress Treatments Using IR Microspectroscopy
Source: Foods. 2021 Aug 11;10(8):1850. doi: 10.3390/foods10081850 (PMC8392388; doi:10.3390/foods10081850)
Supplement: Supplementary file 1 [file foods-10-01850-s001.zip › foods-1301399-supplementary.pdf]

foods

MDPI

Supplementary material

Table S1. Data splitting scheme for the trained and tested microorganisms. The time period after incubation is given in days for each data sets. Each data set consists of 50 spectra. Stress conditions are divided into lifetime conditions, in which the influence is already applied at inoculation and thus active over a life cycle, and sampling condition, in which the influence is a short major stress during sampling.

	Lifetime condition																			
	25 °C				45 °C				2-propanol				NaOH				HCl			
**Data set**	**1**	**2**	**3**	**4**	**1**	**2**	**3**	**4**	**1**	**2**	**3**	**4**	**1**	**2**	**3**	**4**	**1**	**2**	**3**	**4**
B. coag					24	1	2	7					3	6	7	8	3	9	15	17
B. sub	1	6	8	12	20	23	6	27	1	9	14	16	3	6	6	7	3	9	15	17
B. therm	3	6	1	20	6	12	39	40	11	14	14	16	3	6	6	7	10	13	16	17
B. tii	6	9	1	21					2	6	12	14	6	6	7	8	7	10	14	15
E. coli K12	3	6	8	21	20	23	39	40	2	9	14	16	3	6	7	8	6	13	16	17
M. luteus	8	12	20	21	23	6	12	39	1	9	14	16	3	6	6	7	3	9	15	17
Ps. fluor 4358	6	8	20	21					2	9	14	16	6	6	7	8	6	13	15	20
Ps. fluor 50090	6	1	20	21					1	9	14	16	3	6	6	7	6	9	15	17
E. coli TOP10	1	6	20	9	1	6	23	39	3	6	6	7	2	8	14	16	7	10	16	17

	Sampling condition															
	Heat dried				Regular				Desiccator				Cold sampling			
**Data set**	**1**	**2**	**3**	**4**	**1**	**2**	**3**	**4**	**1**	**2**	**3**	**4**	**1**	**2**	**3**	**4**
B. coag	8	9	15	15	8	12	6	8	9	10	13	14	8	12	3	9
B. sub	8	9	15	15	8	12	6	8	9	10	13	14	8	12	2	9
B. therm	14	16	9	15	1	6	7	8	15	21	9	14	14	7	9	10
B. tii	8	9	15	15	8	12	6	8	10	9	10	13	8	12	3	9
E. coli K12	14	16	9	15	1	6	7	12	15	21	9	14	14	20	9	14
M. luteus	9	11	9	15	24	1	2	7	10	1	9	14	9	15	9	13
Ps. fluor 4358	14	16	9	15	1	6	7	8	15	21	9	14	14	20	9	13
Ps. fluor 50090	14	16	9	15	1	6	7	8	15	21	9	14	14	7	9	13
E. coli TOP10	27	7	9	15	1	6	7	12	15	21	9	14	14	20	27	9

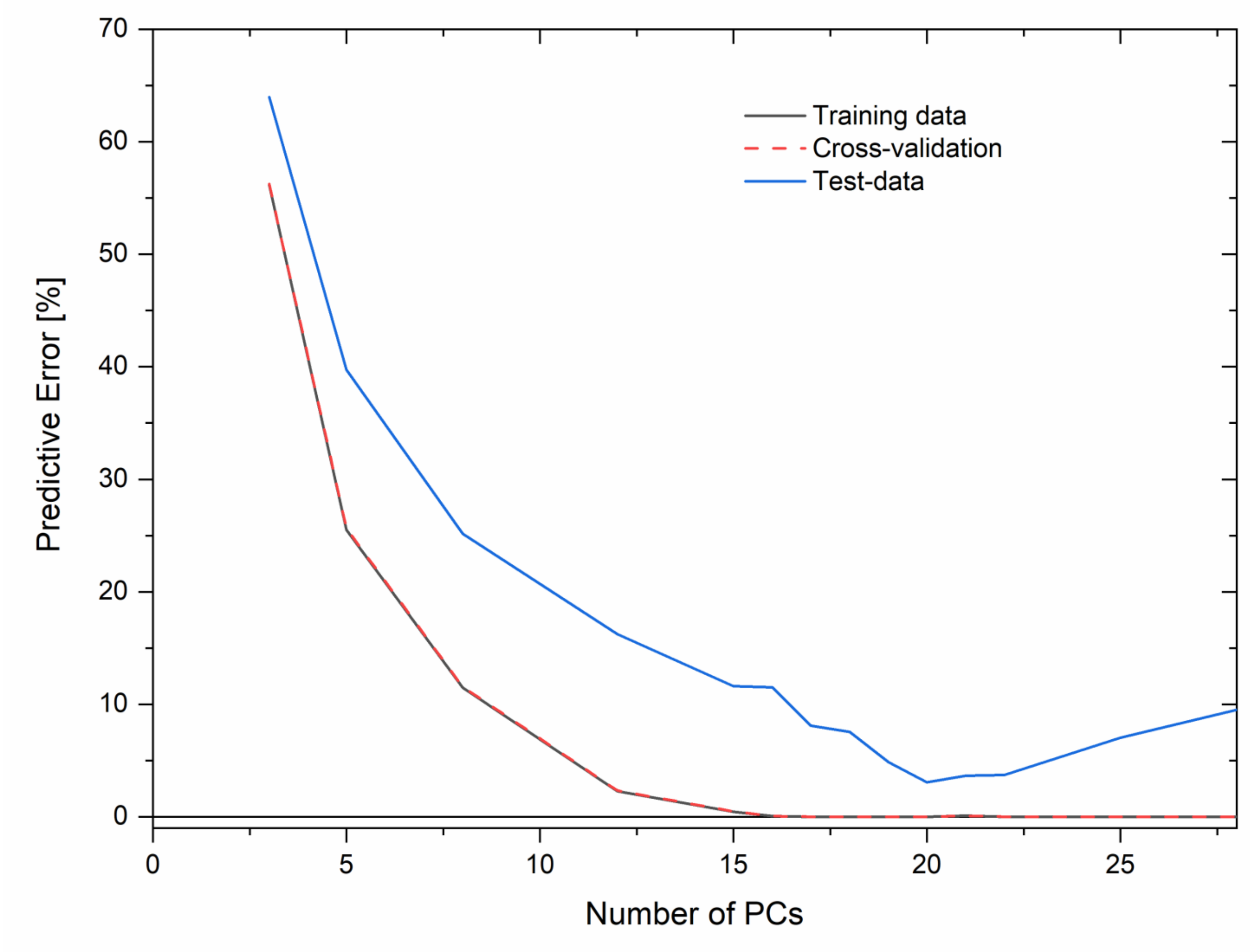

Figure S1. Check for overfitting for the general discrimination of food-related microorganisms.

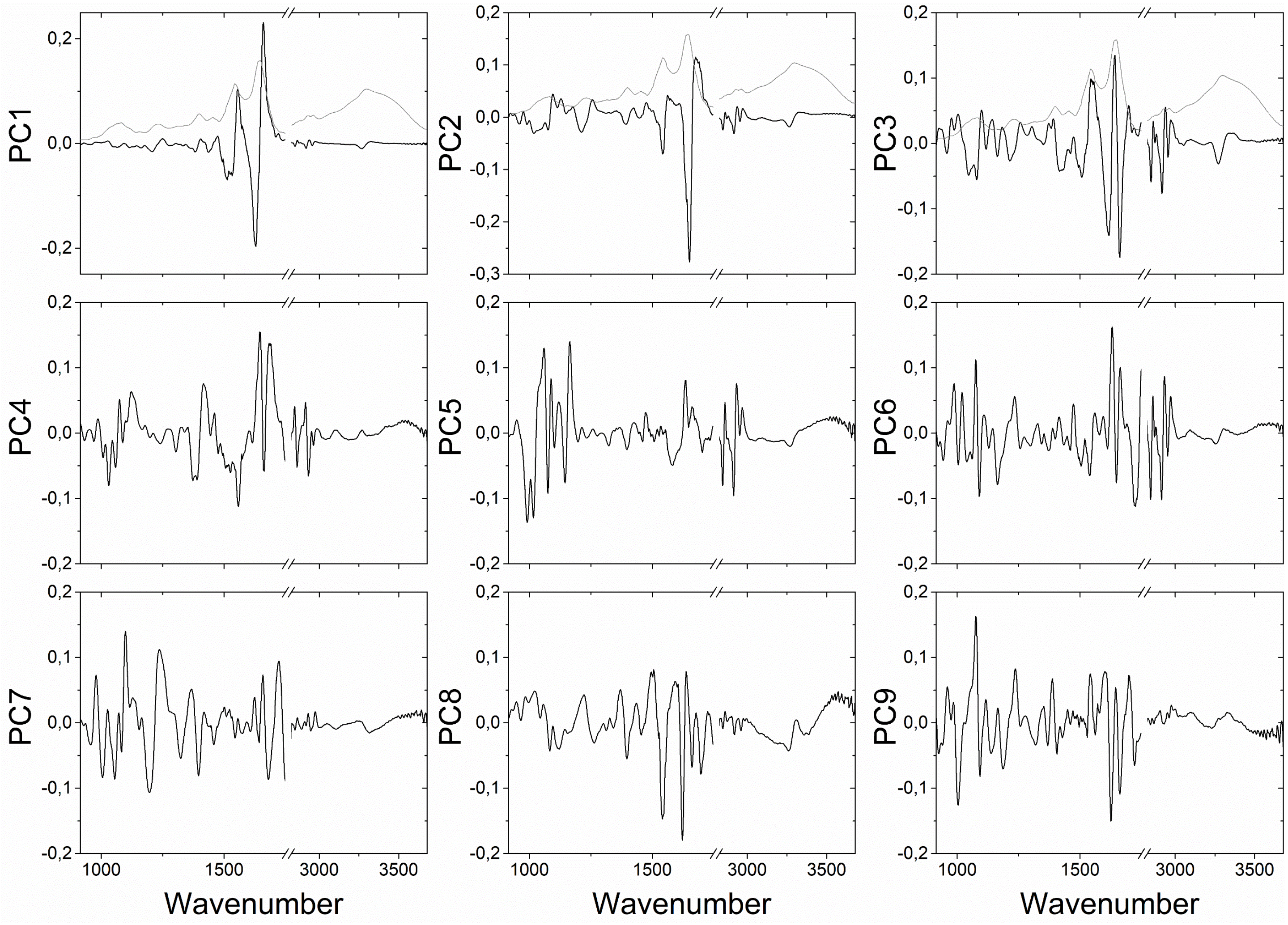

Figure S2. Loadings (PC1 – PC9) of the PCA of the training data set for the bacteria discrimination model. For a better spectral comparison in the graphs for PC1 – PC3 the average spectrum of B. coag is given in gray.

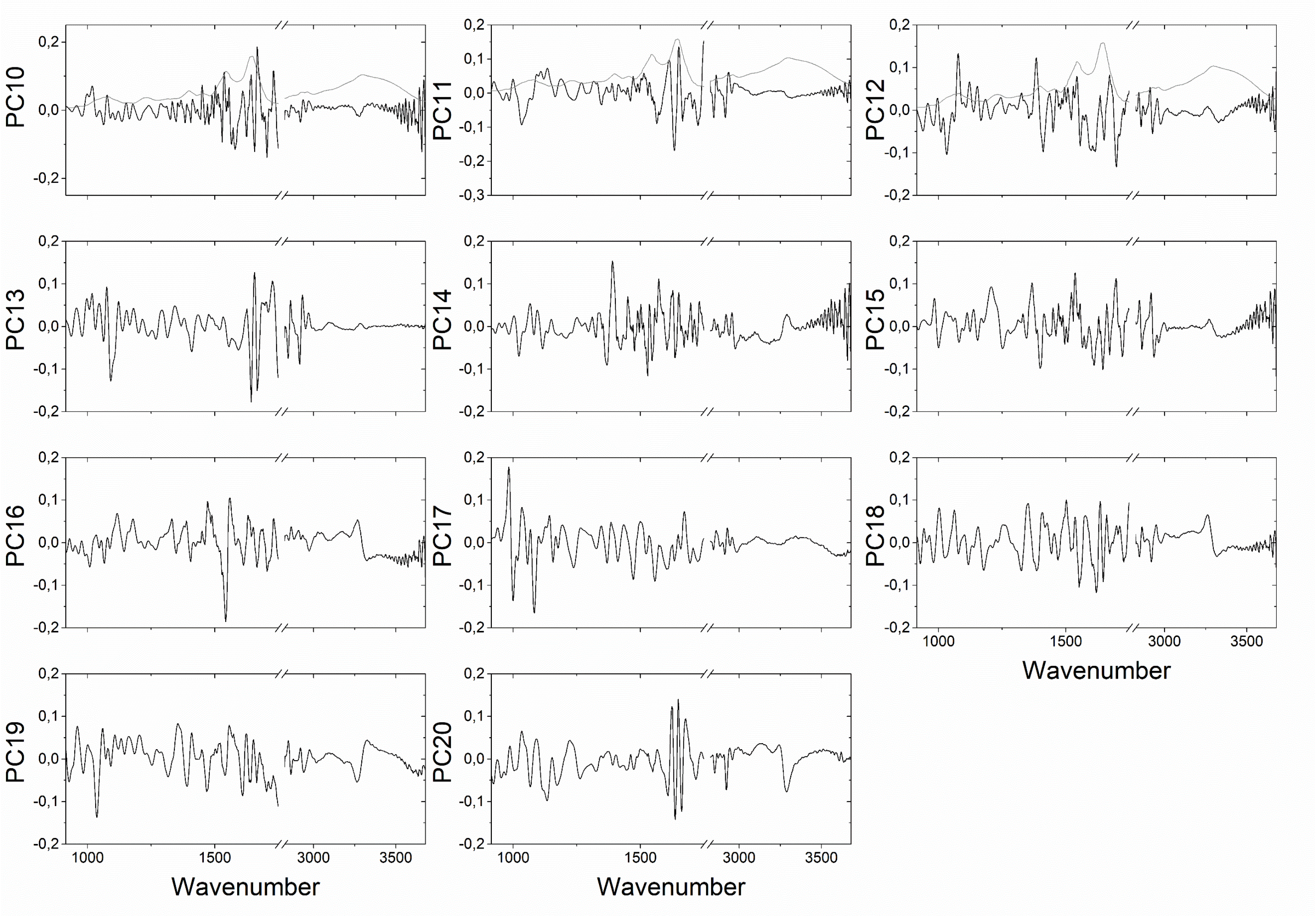

Figure S3. Loadings (PC10 – PC20) of the PCA of the training data set for the bacteria discrimination model. For a better spectral comparison in the graphs for PC10 – PC12 the average spectrum of B. coag is given in gray.

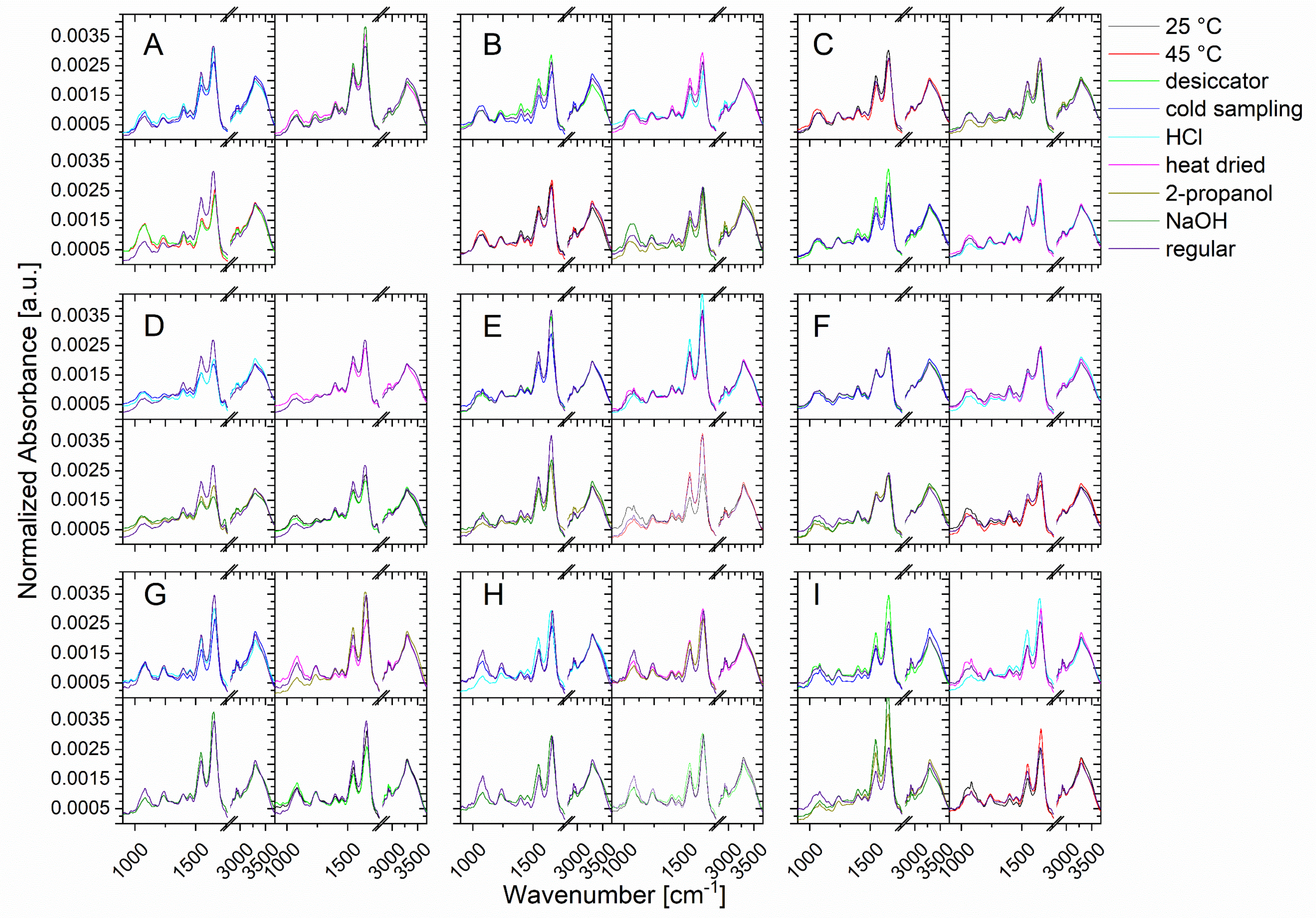

Figure S4. Raw IR spectra of each stress condition vs. regular treated of *B. coag* (A), *B. sub* (B), *B. therm* (C), *B. tii* (D), *E. coli K12* (E), *M. luteus* (F), *Ps. fluor 4358* (G), *Ps. fluor 50090* (***H***) *and E. coli TOP10* (I).

Table S2. Confusion matrix for the independent test data set for the classification of all stress condition for *B. coag*. The values of the table reflect the correct classifications of observations.

B. coag	**Predicted class**							
	**45 °C**	**Desiccator**	**Cold sampling**	**HCl**	**Heat dried**	**2-Propanol**	**NaOH**	**Regular**
45 °C	50	0	0	0	0	0	0	0
Desiccator	0	50	0	0	0	0	0	0
Cold sampling	0	0	50	0	0	0	0	0
HCl	0	0	0	50	0	0	0	0
Heat dried	0	0	0	0	50	0	0	0
2-propanol	0	0	0	0	0	50	0	0
NaOH	0	0	0	0	0	0	50	0
Regular	0	0	0	0	0	0	0	50

Table S3. Confusion matrix for the independent test data set for the classification of all stress condition for *B. sub*. The values of the table reflect the correct classifications of observations.

B. sub	**Predicted class**								
	**25 °C**	**45 °C**	**Desiccator**	**Cold sampling**	**HCl**	**Heat dried**	**2-propanol**	**NaOH**	**Regular**
25 °C	50	0	0	0	0	0	0	0	0
45 °C	0	50	0	0	0	0	0	0	0
Desiccator	0	0	50	0	0	0	0	0	0
Cold sampling	0	0	0	50	0	0	0	0	0
HCl	0	0	0	0	50	0	0	0	0
Heat dried	0	0	0	0	0	50	0	0	0
2-propanol	0	0	0	0	0	0	50	0	0
NaOH	0	0	0	0	0	0	0	50	0
Regular	0	0	0	0	0	0	0	0	50

Table S4. Confusion matrix for the independent test data set for the classification of all stress condition for *B. therm*. The values of the table reflect the correct classifications of observations.

B. therm	**Predicted class**								
	**25 °C**	**45 °C**	**Desiccator**	**Cold sampling**	**HCl**	**Heat dried**	**2-propanol**	**NaOH**	**Regular**
25 °C	50	0	0	0	0	0	0	0	0
45 °C	0	50	0	0	0	0	0	0	0
Desiccator	0	0	50	0	0	0	0	0	0
Cold sampling	0	0	0	50	0	0	0	0	0
HCl	0	0	0	0	50	0	0	0	0
Heat dried	0	0	0	0	0	50	0	0	0
2-propanol	0	0	0	0	0	0	50	0	0
NaOH	0	0	0	0	0	0	0	50	0
Regular	0	0	0	0	0	0	0	0	50

Table S5. Confusion matrix for the independent test data set for the classification of all stress condition for *B. tii*. The values of the table reflect the correct classifications of observations.

B. tii	**Predicted class**							
	**25 °C**	**Desiccator**	**Cold sampling**	**HCl**	**Heat dried**	**2-propanol**	**NaOH**	**Regular**
25 °C	50	0	0	0	0	0	0	0
Desiccator	0	50	0	0	0	0	0	0
Cold sampling	0	0	50	0	0	0	0	0
HCl	0	0	0	50	0	0	0	0
Heat dried	0	0	0	0	50	0	0	0
2-propanol	0	0	0	0	0	50	0	0
NaOH	0	0	0	0	0	0	50	0
Regular	0	0	0	0	0	0	0	50

Table S6. Confusion matrix for the independent test data set for the classification of all stress condition for *E. coli* K12. The values of the table reflect the correct classifications of observations.

E. coli K12	**Predicted class**								
	**25 °C**	**45 °C**	**Desiccator**	**Cold sampling**	**HCl**	**Heat dried**	**2-propanol**	**NaOH**	**Regular**
25 °C	50	0	0	0	0	0	0	0	0
45 °C	0	50	0	0	0	0	0	0	0
Desiccator	0	0	50	0	0	0	0	0	0
Cold sampling	0	0	0	50	0	0	0	0	0
HCl	0	0	0	0	50	0	0	0	0
Heat dried	0	0	0	0	0	50	0	0	0
2-propanol	0	0	0	0	0	0	50	0	0
NaOH	0	0	0	0	0	0	0	50	0
Regular	0	0	0	0	0	0	0	0	50

Table S7. Confusion matrix for the independent test data set for the classification of all stress condition for *M. luteus*. The values of the table reflect the correct classifications of observations.

M. luteus	**Predicted class**								
	**25 °C**	**45 °C**	**Desiccator**	**Cold sampling**	**HCl**	**Heat dried**	**2-propanol**	**NaOH**	**Regular**
25 °C	50	0	0	0	0	0	0	0	0
45 °C	0	50	0	0	0	0	0	0	0
Desiccator	0	0	50	0	0	0	0	0	0
Cold sampling	0	0	0	50	0	0	0	0	0
HCl	0	0	0	0	50	0	0	0	0
Heat dried	0	0	0	0	0	50	0	0	0
2-propanol	0	0	0	0	0	0	50	0	0
NaOH	0	0	0	0	0	0	0	50	0
Regular	0	0	0	0	0	0	0	0	50

Table S8. Confusion matrix for the independent test data set for the classification of all stress condition for *Ps. fluor* 4358. The values of the table reflect the correct classifications of observations.

Ps. fluor 4358	**Predicted class**							
	**25 °C**	**Desiccator**	**Cold sampling**	**HCl**	**Heat dried**	**2-propanol**	**NaOH**	**Regular**
25 °C	50	0	0	0	0	0	0	0
Desiccator	0	50	0	0	0	0	0	0
Cold sampling	0	0	50	0	0	0	0	0
HCl	0	0	0	50	0	0	0	0
Heat dried	0	0	0	0	50	0	0	0
2-propanol	0	0	0	0	0	50	0	0
NaOH	0	0	0	0	0	0	50	0
Regular	0	0	0	0	0	0	0	50

Table S9. Confusion matrix for the independent test data set for the classification of all stress condition for *Ps. fluor* 50090. The values of the table reflect the correct classifications of observations.

***Ps. fluor* 50090**	**Predicted class**							
	**25 °C**	**Desiccator**	**Cold sampling**	**HCl**	**Heat dried**	**2-propanol**	**NaOH**	**Regular**
25 °C	50	0	0	0	0	0	0	0
Desiccator	0	50	0	0	0	0	0	0
Cold sampling	0	0	50	0	0	0	0	0
HCl	0	0	0	50	0	0	0	0
Heat dried	0	0	0	0	50	0	0	0
2-propanol	0	0	0	0	0	50	0	0
NaOH	0	0	0	0	0	0	50	0
Regular	0	0	0	0	0	0	0	50

Table S10. Confusion matrix for the independent test data set for the classification of all stress condition for E. coli TOP10. The values of the table reflect the correct classifications of observations.

**Predicted class**

E. coli TOP10	25 °C	45 °C	Desiccator	Cold sampling	HCl	Heat dried	2-propanol	NaOH	Regular
25 °C	50	0	0	0	0	0	0	0	0
45 °C	0	50	0	0	0	0	0	0	0
Desiccator	0	0	50	0	0	0	0	0	0
Cold sampling	0	0	0	50	0	0	0	0	0
HCl	0	0	0	0	50	0	0	0	0
Heat dried	0	0	0	0	0	50	0	0	0
2-propanol	0	0	0	0	0	0	50	0	0
NaOH	0	0	0	0	0	0	0	50	0
Regular	0	0	0	0	0	0	0	0	50
